# Propolis: A Wonder Bees Product and Its Pharmacological Potentials

**DOI:** 10.1155/2013/308249

**Published:** 2013-12-09

**Authors:** Vijay D. Wagh

**Affiliations:** Department of Pharmaceutics, R. C. Patel Institute of Pharmaceutical Education and Research, Near Karvand Naka, Shirpur, Dist Dhule, Maharashtra 425405, India

## Abstract

Propolis is a natural resinous mixture produced by honey bees from substances collected from parts of plants, buds, and exudates. Due to its waxy nature and mechanical properties, bees use propolis in the construction and repair of their hives for sealing openings and cracks and smoothing out the internal walls and as a protective barrier against external invaders like snakes, lizards, and so forth, or against weathering threats like wind and rain. Bees gather propolis from different plants, in the temperate climate zone mainly from poplar. Current antimicrobial applications of propolis include formulations for cold syndrome (upper respiratory tract infections, common cold, and flu-like infections), wound healing, treatment of burns, acne, herpes simplex and genitalis, and neurodermatitis. Worldwide propolis has a tremendous popularity, but in India the studies over propolis have just started, not extensively reported except few regions of India like Maharashtra, West Bengal, Tamil Nadu, Gujrat, and Madhya Pradesh.

## 1. Introduction

Propolis is a natural resinous mixture produced by honeybees from substances collected from parts of plants, buds, and exudates. The word propolis is derived from Greek, in which pro stands for “at the entrance to” and polis for “community” or “city,” which means this natural product is used in hive defense. Another name of propolis is bee glue. Due to its waxy nature and mechanical properties, bees use propolis in the construction and repair of their hives—for sealing openings and cracks and smoothing out the internal walls [1, 2] and as a protective barrier against external invaders like snakes, lizards, and so forth, or against wind and rain. Bees gather propolis from different plants in different temperate climatic zones.

Honey and propolis provide beneficial effect on human health. Since ancient times propolis has been extensively employed by man, especially in folk medicine to treat several maladies. Egyptians used bee glue to embalm their cadavers as they well knew about its putrefactive properties. Incas employed propolis as an antipyretic agent. Greek and Roman physicians used it as mouth disinfectant and as an antiseptic and healing product in wound treatment, prescribed for topical therapy of cutaneous and mucosal wounds [2]. Propolis was listed as an official drug in the London pharmacopoeias of the 17th century. Due to its antibacterial activity, in Europe propolis became very popular between the 17th and 20th centuries. In Italy bee glue was used as a violin varnish [3] by Stradivari. In the end of the 19th century, propolis was widely used due to its healing properties and in the Second World War it was employed in several Soviet clinics for tuberculosis treatment, due to the observed decline of lung problems and appetite recovery. In the Balkan states propolis was applied to treat wounds and burns, sore throat, and stomach ulcer [4]. The first scientific work with propolis was published in 1908 including its chemical properties and composition which was further indexed to chemical abstract [5].

Nowadays, propolis is a natural remedy found in many health food stores in different forms for topical use. It is also used in cosmetics or as popular alternative medicine for self-treatment of various diseases. Current applications of propolis include formulations for cold syndrome (upper respiratory tract infections, common cold, and flu-like infections), as well as dermatological preparations useful in wound healing, treatment of burns, acne, herpes simplex and genitalis, and neurodermatitis. Propolis is also used in mouthwashes and toothpastes to prevent caries and to treat gingivitis and stomatitis. It is widely used in cosmetics and in health foods and beverages. It is commercially available in the form of capsules, mouthwash solutions, creams, throat lozenges, powder, and also in many purified products from which the wax was removed. Due to its antimicrobial, antiviral, and antioxidant properties, it is widely used in human and veterinary medicine, pharmacology, and cosmetics.

## 2. Propolis Characteristics, Source, and Bioactive Composition

### 2.1. Characteristics

Propolis is a lipophilic in nature, hard and brittle material and it becomes soft, pliable, gummy, and very sticky when heated [6]. It possesses a characteristic and pleasant aromatic smell and varies in color from yellow green to red and to dark brown depending on its source and age [2–7]. Depending on the origin of the resins, it also ranges from yellow to dark brown. But even transparent propolis has been reported.

### 2.2. Composition

Propolis is a complex mixture made by bee-released and plant-derived compounds. In general, raw propolis is composed of around 50% resins, 30% waxes, 10% essential oils, 5% pollen, and 5% of various organic compounds [1, 8, 9]. More than 300 constituents were identified in different samples [7–10] and new ones are still being recognized during chemical characterization of new types of propolis [2, 11, 12]. The proportions of the various substances present in the propolis depend upon its place and time of collection.

Many analytical methods have been used for separation and identification of propolis constituents and the substances identified belong to the following groups of chemically similar compounds: polyphenols; benzoic acids and derivatives; cinnamic alcohol and cinnamic acid and its derivatives; sesquiterpene and triterpene hydrocarbons; benzaldehyde derivatives; other acids and respective derivatives; alcohols, ketones, and heteroaromatic compounds; terpene and sesquiterpene alcohols and their derivatives; aliphatic hydrocarbons; minerals; sterols and steroid hydrocarbons; sugars and amino acids [13]. As it may be expected, volatile compounds (produced by the source plants) are present in low amounts [10]. Sugars are thought to be introduced accidentally during the elaboration of propolis and/or passage of bees over the resin. Some compounds are common in all propolis samples and determine its characteristics properties.

Propolis of different origin contains different constituents. Some constituents are present in many samples from different places. Some constituents are present in sample from specific plant origin [14].

Different geographical origin of propolis sample varies with its biological activity due to different climatic conditions [15]. The essential principal compounds responsible for biological activities are polyphenols, aromatic acids, and diterpenic acids, but very few different propolis types have been different in their main bioactive compounds (Table 1). Different composition is also related to specific flora of the region and treatments of raw material.

### 2.3. Melting Point

Propolis is soft, pliable, and sticky substance at 25°C to 45°C. Particularly, in frozen condition, it becomes hard and brittle. It will remain brittle after such treatment even at higher temperatures. Above 45°C, it will become increasingly sticky and gummy. Propolis will become liquid at 60°C to 70°C, but for some samples the melting point may be as high as 100°C.

### 2.4. Solubility of Propolis

Considering the complex structure of propolis, it cannot be used directly. Propolis is extracted commercially with suitable solvent. The most common solvents used for extraction are water, methanol, ethanol, chloroform, dichloromethane, ether, and acetone. Many of the bactericidal components are soluble in water or alcohol [16] which should remove the inert material and preserve the desired compounds. Propolis composition depends upon the geographical region and second one the method of extraction [7], the solvent should be carefully chosen [17]. The main solvents used for extraction of bioactive compounds and other chemical compounds extracted are determined in Tables 2 and 3.

### 2.5. Propolis in Indian Scenario

India, being a vast country, has a number of varieties of propolis differing in chemical compositions and medicinal values which are mentioned in Tables 2 and 3. But unfortunately it is still to be explored.

### 2.6. Antioxidant Activity

To the best of our knowledge, this is the first report published on the antioxidant activity of Indian propolis extract and its chemical constituent's pinocembrin and galangin. In all the antioxidant assay systems, aqueous extract of propolis (AEP) showed higher activity compared to the ethanolic extract of propolis (EEP). This may be due to its higher polyphenols content. So, AEP can be a good substitute of ethanol extract. Moreover, it can be used in prevention of various free radical related diseases. Galangin also showed comparable activity with that of AEP and EEP and highest activity than pinocembrin. This is due to structural differences between these two compounds. Further research is underway to analyze the constituents of AEP and their antioxidant activity [18].

Roy et al. extended pinocembrin and galangin in the rapid synthesis of stable Ag and Au nanoparticles having wide spectrum of fascinating morphologies. Both of these two extracts were found to be extremely efficient in the synthesis of Ag and Au nanoparticles under alkaline condition for a given metal ion precursor; the kinetics of a particle synthesis were remarkably similar in all the cases, as it is evident from the absorption spectra monitored over time [19].

The free radical scavenging effect of propolis as well as of vitamin C in 1,1-diphenyl-2-picrylhydrazyl (DPPH) free radical system was determined. The free radical scavenging activity of EEP was 70.96% and 72.97%, respectively, in the concentration range of 100 mcg at the difference of 30 min and 1 hr, respectively. The result of free radical scavenging effect of vitamin C was 94.7% at 100 mcg and 93.4% at 10 mcg [16].

### 2.7. Antibacterial Activity

According to Kumar et al., the antimicrobial property of propolis collected from Gujarat by agar diffusion method against *Staphylococcus aureus*, *Bacillus subtilis*, *Pseudomonas aeruginosa*, *Escherichia coli*, *Candida albicans,* and *Asparagus nigar*. Ethanolic extracts of sample (conc. 200 mg/mL) showed high antibacterial activity against Gram-positive, that is, *Bacillus subtilis,* but least activity against Gram-negative bacteria (*P*. *aeruginosa* and *E*. *coli*). The yeast *C*. *albicans* showed the moderate zone of inhibition whereas *A*. *Niger* did not show any activity. However, the least was in the 40% methanolic extracts [16].

Selvan et al. collected propolis from different places in Hunasamaranahalli Village near Bangalore. they observed that bee propolis in combination with chlorhexidine possesses high antimicrobial activity against *Streptococcus mutans*. Propolis in combination with chlorhexidine can suppress the pathogenic potentials of a dental plaque by inhibiting the adherence and accumulation of cariogenic Streptococci on the tooth surface. The inhibition of the growth of the clinical stress by trace quantities of a propolis suggests that it can be used in the treatment of dental caries.

### 2.8. Biological Activities

The use of different solvents changes activity of main biologically active constituent in propolis. Those are responsible for its many biological properties and also change by geographic origin and dosage form [20]. Presence of flavonoids and phenolic esters propolis is responsible for its potential effects with specific reagent.

### 2.9. Antifungal Activity

Propolis has shown fungicide effects on juice spoilage fungi *Candida famata, C. glabrata, C. kefyr, C. pelliculosa, C. parapsilosis, *and* Pichia ohmeri* [21]; the fungicidal effect was associated with the presence of flavonoids [22]. Propolis is the bee product with the highest antifungal activity as tested with 40 yeast strains of *C. albicans, C. glabrata, C. krusei, *and* Trichosporon *spp. [23]. Propolis inhibited the growth *C. albicans *(MIC 0.2–3.75 *μ*g/mL), *C. glabrata* (MIC 0.03–7.5 *μ*g/mL), *Trichosporon *spp. (MIC 0.1–0.4 *μ*g/mL), and *Rhodotorula *sp. (MIC <0.01 *μ*g/mL) and the most sensitive strain was *Rhodotorula *spp. The most resistant strain was *C. Albicans*. In an unpublished study in Bangalore, Indian propolis has been observed to be more effective than routinely used anticaries agents in inhibiting the growth of *Streptococcus mutans* which is a frequent cause of dental caries [24]. Oliveira et al. (2006) was studied the 67 samples of yeasts isolated and identified from samples of onychomycosis comprising the following species: *Candida albicans*, *Candida parapsilosis*, *Candida tropicalis*, *Candida kefyr*, *Candida guilliermondii*, *Candida lusitaniae*, *Candida glabrata*, *Candida stellatoidea*, *Candida Trichosporon* sp. including *T*. *asahii*, *T*. *ovoides,* and *T*. *cutaneum*, one *Geotrichum candidum,* and three *Saccharomyces cerevisiae*. *Trichosporon* sp. was the most sensitive species, showing MIC_50_ and MIC_90_ of 1.25 × 10^−2 ^mg/mL of flavonoids, and *Candida tropicalis* was the most resistant, with CFM_50_ of 5 × 10^−2 ^mg/mL of flavonoids and MFC_90_ of 10 × 10^−2 ^mg/mL [25]. The activity of ethanolic extraction of propolis was elevated by disc diffusion method when the concentration increased to 20% and 30%. EEP was not effective against *C*. *albicans* [26].

### 2.10. Vaginal Use

To formulate the propolis microparticles (PMs) from Brazilian propolis [27, 28] and to check activity of the propolis extract (PE) against clinical yeast *C. albicans* and 31 non-*C. albicans* (*C. glabrata*, *C. tropicalis*, *C. guilliermondii,* and *C. parapsilosis*) isolates of importance in the vulvovaginal candidiasis (VVC). Moreover, the main antifungal drugs used in the treatment of VVC were also tested. *C. albicans* isolates showed resistance or dose-dependent susceptibility for the azolic drugs and Amphotericin B. Non-*C. albicans* isolates showed more resistance and dose-dependent susceptibility for the azolic drugs than *C. albicans*. However, all of them were sensitive or dose-dependent susceptible for Amphotericin B. All yeasts were inhibited by PE and PMs, with small variation, independent of the species of yeast. The overall results provided important information for the potential application of PMs in the therapy of VVC and the possible prevention of the occurrence of new symptomatic episodes [27].

### 2.11. Antibacterial Activity

The disc diffusion method is one of the most popular methods used to determine the antimicrobial activity. A suspension of a sensitive indicator microorganism is inoculated on agar plates by spreading homogeneously on its surface, and blank paper discs containing the sample to be checked for antimicrobial activity are placed on top. After an incubation period at optimal temperature, antibacterial activity is evaluated by determining the diameter of the growth inhibition zones in the agar layer surrounding the disc [15]. Some authors argue that this laborious method is unreliable for comparing bioactivities, as results are influenced by the solubility and hence the diffusivity of the individual constituents in agar, proposing the use of another methodology which is also commonly used for the same purpose, the dilution method. In this procedure, propolis samples are serially twofold diluted and a fixed volume is added to liquid or solid medium, by making a series of concentrations. Bacterial inoculums are added to each experimental condition and the occurrence of growth is analyzed after incubation at optimal conditions. Broth microdilution is considered a good method for a rapid and simultaneous screening of multiple samples; for comparing propolis extracts and giving more consistent results, it is suitable method to be used. Additionally, it allows the determination of the minimal inhibitory concentration (MIC) and the minimal bactericidal concentration (MBC) which are, respectively, the lowest concentration that inhibits visible bacterial growth and the lowest concentration that kills bacteria [16–35]. Briefly, thin-layer chromatography plates where propolis samples were eluted are covered with agar suspensions of the microorganism whose sensitivity is going to be tested. Antibacterial activity is visualized as clear areas after proper incubation [36].

Data from studies concerning antibacterial properties of propolis support the fact that propolis is active mainly against Gram-positive bacteria in and shows lower activity against the Gram-negative ones at small quantity or is inactive at all [7, 35, 37–41]. Such results can be seen in the work of Kujumgiev et al. (1999) who tested propolis samples from different geographic regions (tropical and temperate zones) against *Staphylococcus aureus *and *Escherichia coli*. All the extracts displayed significant antibacterial activity against *S*. *aureus* but none was active against *E. coli*; it is also relevant that all the 12 samples tested, from different origins, showed the same effect.

Several studies have been performed to evaluate this property against Gram-positive and Gram-negative bacteria (Table 4) collected from laboratory or isolated from clinical samples using different types of propolis by using different approaches.

The activity of ethanolic extract of Al-Museiab propolis (EEMP) against *E. coli*, *Salmonella typhi*, *Listeria monocytogenes*, *Helicobacter pylori*, *Streptococcus pyogenes, Pseudomonas aeruginosa*, *Staphylococcus aureus*, *Klebsiella pneumonia*, and *Enterobacter aerogenes* isolates by the method of disc diffusion and agar-well diffusion; *Staphylococcus aureus* was highly sensitive to EEMP than other Gram positive and Gram-negative bacteria, while standard *E. coli* strain was highly sensitive to EEMP than other Gram-negative bacteria. Results of disc diffusion methods of crude EEMP at 10% concentration showed that *S. Aureus* was highly sensitive to EEMP inhibition while *C. albicans* was resistant. Statistical analysis showed significant differences (*P* ≤ 0.05) between results of disc and agar diffusion methods of EEP at concentration of 10%, while there were no significant differences (*P* ≤ 0.05) at concentrations of 20% and 30% of extract, respectively [26].

Furth Grange and Davey reported that ethanol extracts from propolis (EEP) completely inhibited the growth of *S. aureus, Enterococcus *spp., and *Bacillus cereus*, partially inhibited *Pseudomonas aeruginosa* and *E. coli* growth, and had no effect on *Klebsiella pneumoniae* [29]. The mechanism of antimicrobial activity of propolis is complex and could be attributed to the synergistic activity between phenolic and other compounds [42], mainly to the flavonoids pinocembrin, galangin, and pinobanksin [43]. A stronger activity was observed on gram-positive bacteria growth [1]. The antimicrobial activity was observed on *Staphylococcus aureus* [44, 45], *Streptococcus pyogenes* [46], Gram-positive and Gram-negative bacteria species and *Candida* [37], *Streptococcus mutans* [24]; anaerobic bacteria of human oral cavity [47], *Salmonella* [48], and on miscellaneous microorganisms including *Mycobacterium* [49] In screening studies at a dilution of 1 : 20 (3 mg of solid material per mL) in nutrient agar, Thus it appeared to have a preferential inhibitory effect on cocci and Gram-positive rods [29].

### 2.12. Antiprotozoan Activity

Antiprotozoal activity is evaluated by an in vitro growth inhibitory effect on a culture of parasites after incubation in the presence of different concentrations of propolis. The effect of European propolis on protozoa reported by several publications that cause diseases in humans and animals such as trichomoniasis, toxoplasmosis, giardiasis, Chagas disease, leishmaniasis, and malaria. Indeed, antiprotozoan activity has also been reported on *Giardia lamblia, Trichomonas vaginalis, Toxoplasma gondii, Leishmania donovani, *and* Trypanosoma cruzi* [50, 51]. Also an antiprotozoan activity of EEP was reported against *G. duodenalis* [52].

### 2.13. Antioxidant Activity

Propolis is notable for its antioxidant properties. The antioxidants present in propolis [53, 54] play a great role in its immunomodulatory properties [55]. The flavonoids concentrated in propolis are powerful antioxidants. One of the most commonly used techniques to evaluate antioxidant potential is based on the depletion of free radicals by the addition of scavenger compounds. Measurements of 1,1-diphenyl-2-picrylhydrazyl (DPPH) radical consumption are related to the intrinsic ability of a substance or a complex mixture to donate hydrogen atoms or electrons to this reactive species in a homogeneous system. It was reported that propolis increases the cellular immune response through the increase of mRNA for interferon-*γ* and activates the production of cytokines [56].

Water extracts of propolis collected from the three geographical regions (Motobes, Kafr El-Sheikh, and Desouk) in Kafr El-Sheikh Governorate, Egypt, were prepared. The extracts were analyzed for the determination of total polyphenols which ranged from 5.70 to 8.79 g/100 gm of the sample and from 22.80 to 34.30 g/100 g of the freeze drier extract. The total flavonoids content ranged from 3.05 to 4.85 g/100 g of the sample. Water extracts of propolis were evaluated for antioxidant activity using *β*-carotene bleaching and 1,1-diphenyl-2-picrylhydrazyl (DPPH) free radical scavenging assay system. It was observed that all propolis had strong antioxidant activity due to their contents of total phenols and flavonoids. The highest activity was found for the sample from Desouk followed by these from Kafr El-Sheikh, then those from Motobes. Freeze-dried extract of propolis can be used as natural antioxidant in sunflower oil as compared to BHT and TBHQ. Propolis from Desouk and Kafr El-Sheikh at 200 and 300 ppm were similar in reducing peroxide values and both of them at 300 ppm were better than BHT but lower than TBHQ, added at 200 ppm concentration, in reducing peroxides and hydroperoxidase production in sunflower oil at 63°C for 4 days [57].

Antioxidants have been shown to be capable of scavenging free radicals and thereby protecting lipids and other compounds such as vitamin C from being oxidized or destroyed [58]. It is probable that active free radicals, together with other factors, are responsible for cellular aging and degradation in such conditions as cardiovascular diseases, arthritis, cancer, diabetes, Parkinson's disease, and Alzheimer's disease. Oxidative damage may also result in poor liver function. Studies on rats in vitro show that propolis extracts protect against damage to liver cells [59].

The sample from Algarve, south of Portugal region, contains phenolic compound and this phenolic group shows antioxidant activity. Water was revealed to be less effective and less toxic for extracting phenolic compounds from propolis than the methanol and water/ethanol. In spring, higher amounts of phenols (total phenols, flavones, flavonols, flavanones, and dihydroflavonols) were detected in hydroalcoholic extracts of propolis than in winter [60]. According to zones, the levels of phenols changed. In spring, higher amounts of phenols were detected in hydroalcoholic extracts of propolis than in winter. Among the three main areas of Algarve where samples were collected, those from Barrocal had the highest levels of polyphenols, depending on the season (winter or spring). Within each area, the levels of phenols changed according to the zone. Concerning antioxidant activity, samples from Barrocal presented better radical scavenging abilities than those from the remaining areas, depending on the antioxidant method and collection season [60]. Miguel et al. extended their work and described antioxidant property which was evaluated along with the capacity of extracts of propolis for scavenging DPPH [61] and ABTS free radicals as well as superoxide anion [62].

### 2.14. Antitumoral Activity 

The antitumor activity of propolis was reviewed by Orsolic et al. The chemopreventive activity of propolis in animal models and cell cultures is likely to be the result of their ability to inhibit DNA synthesis in tumour cells, their capability to induce apoptosis of tumour cells, and their property to activate macrophages to produce factors capable of regulating the function of B, T and NK cells, respectively. Moreover, these results suggest that flavonoids from propolis play a protective role against the toxicity of the chemotherapeutic agents or radiation in mice, giving hope that they may have similar protective action in humans. The combination with an adjuvant antioxidant therapy may enhance the effectiveness of chemotherapy by ameliorating the side effect on leukocytes, liver, and kidneys and consequently enabling dose escalation [63]. Although many polyphenols have an antimetastatic activity, caffeic acid phenethyl esters (CAPE) from poplar propolis and Artepillin C from *Baccharis* propolis have been identified as the most potent antitumor agents [64–68].

The in vitro anticarcinogenic potential of propolis in human lymphocytes was investigated. Blood samples were obtained from ten healthy males, nonsmoking volunteers, which were incubated and exposed to increasing concentrations of propolis (0.01, 0.05, 0.1, 0.2, 0.5, 0.7, and  1.0 mL). The mean micronucleus rates were 1.4770.38–4.0270.64. Mitotic index rates were between 19.4572.22 and 0.2870.33. The differences between the control and exposed cells were statistically significant (pp 0 : 05). Exposure to different concentrations of propolis cannot produce a carcinogenic effect in peripheral human lymphocytes in vitro. However, increasing micronucleus (MN) rates showed that propolis could have a carcinogenic effect in high concentrations [69].

### 2.15. Anti-Inflammatory Activity

Inflammation is the complex biological response of vascular tissues to harmful stimuli, such as pathogens, damaged cells, irritants, and free radicals. Anti-inflammatory activity means the primary effect of the host defense system. The anti-inflammatory activity of propolis has been reviewed by Almeida and Menezes. Propolis has inhibitory effects on myeloperoxidase activity, NADPH-oxidase ornithine decarboxylase, tirosine-protein-kinase, and hyaluronidase from guinea pig mast cells. This anti-inflammatory activity can be explained by the presence of active flavonoids and cinnamic acid derivatives. The former includes acacetin, quercetin, and naringenin; the latter includes caffeic acid phenyl ester (CAPE) and caffeic acid (CA) [70]. CAPE and galangin, both being typical poplar propolis constituents, exhibited anti-inflammatory activity and significantly inhibited carrageenan oedema, carrageenan pleurisy, and adjuvant arthritis inflammations in rats [71, 72]. An ethanol extract of propolis suppressed prostaglandin and leukotriene generation by mouse peritoneal macrophages in vitro and during zymosan-induced acute peritoneal inflammation in vivo. Dietary propolis significantly suppressed the lipoxygenase pathway of arachidonic acid metabolism during inflammation in vivo. CAPE was a more potent modulator of arachidonic acid metabolism than caffeic acid, quercetin, and naringenin [73].

### 2.16. Hepatoprotective Activity

Protective potential of propolis was evaluated against mercury-induced oxidative stress and antioxidant enzymatic alteration in mice liver. Exposure to mercuric chloride (HgCl_2_; 5 mg/kg; i.p.) induced oxidative stress by increasing lipid peroxidation and oxidized glutathione level along with concomitant decrease in glutathione and various antioxidant enzymes. Mercury intoxication deviated the activity of liver marker enzyme in serum. Conjoint treatment of propolis (200 mg/kg; p.o.) inhibited lipid peroxidation and oxidized glutathione level whereas increased glutathione level. Activity of antioxidants enzymes, that is, superoxide dismutase, catalase, glutathione S-transferase, and glucose 6-phosphate dehydrogenase, was also restored concomitantly toward control after propolis administration. Release of serum transaminases alkaline phosphatase, lactate dehydrogenase, and *γ*-glutamyl transpeptidase was significantly restored toward control after propolis treatment. Results suggest that propolis augments the antioxidant defense against mercury-induced toxicity and provides evidence that it has therapeutic potential as hepatoprotective agent [59].

Bhadauria et al.'s study had been conducted to confirm the protective role of propolis extract in CCl_4_-induced hepatorenal oxidative stress and resultant injury. Propolis extracts collected from Gwalior district and 24 female Sprague Dawley rats were used for experiment. Animals were exposed to CCl 4 (0.15 mL/kg, i.p.) for 12 weeks (5 days/week) followed by treatment with propolis extract (200 mg/kg, p.o.) for consecutive 2 weeks. Ethanolic extract of propolis successfully prevented these alterations in experimental animals. Activities of catalase, adenosine triphosphatase, glucose-6-phosphatase, acid, and alkaline phosphatase were also maintained towards normal with propolis therapy. Light microscopical studies showed considerable protection in liver and kidney with propolis treatment and, thus, substantiated biochemical observations. This study confirmed hepatoprotective potential of propolis extract against chronic injury induced by CCl_4_ by regulating antioxidative defense activities [33].

### 2.17. Antidiabetic Activity

The effect of ethanolic extract of propolis against experimental diabetes mellitus-associated changes was examined. Diabetes was induced experimentally in rats by i.p. injection of streptozotocin (STZ) in a dose of 60 mg/kg bwt for 3 successive days. Blood urea nitrogen (BNU), creatinine, glucose, lipid profile, malondialdehyde (MDA), and urinary albumin were measured. Superoxide dismutase (SOD), glutathione (GSH), catalase (CAT), and MDA were measured in the renal tissue. The results showed decreased body weight and increased kidney weight in diabetic animals. Compared to the control normal rats, diabetic rats had higher blood glucose, BNU, creatinine, total cholesterol, triglycerides, low-density lipoprotein-cholesterol (LDL-C), MDA and urinary albumin, and lower high-density lipoprotein-cholesterol (HDL-C) levels. Moreover, renal tissue MDA was markedly increased while SOD, GSH, and CAT were significantly decreased. Oral administration of propolis extract in doses of 100, 200, and 300 mg/kg bwt improved the body and kidney weights, serum glucose, lipid profile, MDA, and renal function tests. Renal GSH, SOD, and CAT were significantly increased while MDA was markedly reduced. These results may suggest a strong antioxidant effect of propolis which can ameliorate oxidative stress and delay the occurrence of diabetic nephropathy in diabetes mellitus [74].

The effect of Chinese and Brazilian propolis on streptozotocin-induced type 1 diabetes mellitus in Sprague Dawley rats was studied [75]. The results showed that Chinese propolis and Brazilian propolis significantly inhibited body weight loss and blood glucose increase in diabetic rats. In addition, Chinese propolis-treated rats showed an 8.4% reduction of glycated hemoglobin levels compared with untreated diabetic rats. Measurement of blood lipid metabolism showed dyslipidemia in diabetic rats and Chinese propolis helped to reduce total cholesterol level by 16.6%. Moreover, oxidative stress in blood, liver, and kidney was improved to various degrees by both Chinese propolis and Brazilian propolis. An apparent reduction in levels of alanine transaminase, aspartate transaminases, and blood urea nitrogen and urine microalbuminuria excretion rate demonstrated the beneficial effects of propolis on hepatorenal function.

### 2.18. Immunomodulatory Action

The immunomodulatory action of a water-soluble derivative (WSD) of natural propolis was investigated. The oral and parenteral administration of the WSD enhanced the survival rate and the mean survival time in experimental bacterial (*Klebsiella pneumoniae*, *Staphylococcus aureus*) and fungal (*Candida albicans*) infections in mice. An increased resistance was observed also in *Klebsiella pneumoniae* infection induced after cyclophosphamide treatment. The WSD stimulated peritoneal macrophages to produce in vitro interleukin-1, which corresponded to their elevated total protein secretion. In addition, WSD failed to trigger lymphocyte proliferation as determined by popliteal lymph node assay. The WSD was suggested to augment nonspecific host defense via macrophage activation [76].

### 2.19. Dental Action

The antimicrobial activity of five propolis samples collected from four different regions in Turkey and from Brazil against nine anaerobic (*Peptostreptococcus anaerobius*, *Peptostreptococcus micros*, *Prevotella oralis*, *Prevotella melaninogenica*, *Porphyromonas gingivalis*, *Fusobacterium nucleatum*, *Veillonella parvula*, *Lactobacillus acidophilus*, and *Actinomyces naeslundii*) strains was evaluated and determined minimum inhibitory concentrations (MIC) and minimum bactericidal concentrations (MBC) of EEP on the growth of test microorganisms by using agar dilution method. All strains were susceptible and MIC values ranged from 4 to 512 mg/mL for propolis activity. Propolis from Kazan-Ankara showed most effective MIC values to the studied microorganisms. MBC values of Kazan-Ankara EEP samples ranged from 8 to 512 mg/mL. Death was observed within 4 h of incubation for *Peptostreptococcus anaerobius* and *micros* and *Lactobacillus acidophilus* and *Actinomyces naeslundii*, while being 8 h for *Prevotella oralis*, *Prevotella melaninogenica,* and *Porphyromonas gingivalis*, 12 h for *Fusobacterium nucleatum*, and 16 h for *Veillonella parvula*. It was shown that propolis samples were more effective against Gram-positive anaerobic bacteria than Gram-negative ones. Propolis is used in oral cavity diseases as it contains flavonoids such as pinobanksin, quercetin, naringenin, galangine, chrysin, and aromatic acids such as caffeic acid determined by GC-MS analysis [77].

Dental caries is an infectious disease of worldwide public health concern. Among the bacteria involved in this pathology are *Streptococcus mutans*, *Streptococcus sobrinus,* and organisms belonging to the genera *Actinomyces* and *Lactobacillus*. The pharmaceutical industry is focusing on the discovery of new antibacterial products after a greater resistance to those already known. Effect of ethanolic propolis extracts on the bacterium *Lactobacillus fermentum* was studied. This bacterium was isolated after its identification by polymerase chain reaction using species-specific primers and after growing microbiological samples from cavities of patients diagnosed with dental caries and with indication of tooth extraction. *L*. *fermentum* was detected in 9 of 40 patients, corresponding to 22%. The susceptibility study, carried out by microplate dilution, found antimicrobial activity of ethanolic extract of propolis. Among the results, it was noticed that these polyphenols showed concentrations ranging between 9 ± 0.3 and 85 ± 2.1 mg/mL. The chromatographic analysis allowed the identification of caffeic acid, myricetin, quercetin, kaempferol, apigenin, pinocembrin, galangin, and caffeic acid phenethyl ester [78].

## 3. Allergy, Rhinitis, and Asthma

No side effects were related in mice, rats, and humans after Brazilian green propolis administration [79–82]. Propolis is nontoxic, and the safe concentration for humans would be approximately 1.4 mg/kg and day or 70 mg/day. However, cases of allergy and contact dermatitis to propolis have been always reported [81], mainly among bee keepers [83, 84]. Rajpara et al. mentioned that the increased incidence of contact dermatitis over the last two decades is likely due to its use in cosmetic and pharmaceutical preparations.

Rhinitis is a symptomatic disorder of the nose, with nasal obstruction, secretion, and sneezing, most commonly induced by allergen exposure, bacteria, or virus. It is a global health problem, affecting social life, sleep, school and work performance, regardless of gender, age, and ethnic background [85]. Shinmei et al. (2009) studied the effect of Brazilian propolis on sneezing and nasal rubbing in experimental allergic rhinitis of mice, concluding that propolis may be effective in the relief of symptoms of allergic rhinitis through inhibition of histamine release. A single administration of propolis caused no significant effect on both antigen-induced nasal rubbing and sneezing at a dose of 1000 mg/kg, but a significant inhibition was observed after repeated administration for 2 weeks at this dose. Asthma is a chronic inflammatory disorder of the pulmonary airways due to the hyperresponsiveness to inhaled allergens, leading to reversible airflow obstruction and airway inflammation, persistent airway hyperactivity, and airway remodeling [86]. Khayyal et al. (2003) administered an aqueous extract of propolis 13% daily for 2 months to patients with mild-to-moderate asthma. As a result, propolis-treated patients showed a reduced incidence and severity of nocturnal attacks and improvement of ventilatory functions, which was associated with decreased prostaglandins, leukotriene, and proinflammatory cytokines (TNF-, IL-6, and IL-8) and increased IL-10. CAPE (10 mg/kg/day) attenuated allergic airway inflammation and hyperresponsiveness in a murine model of ovalbumin-induced asthma [87]. It was reported that the oxidative stress may have a crucial role in the pathogenesis of bronchial asthma, and Cape may be useful as an adjuvant therapy for its treatment.

## 4. Formulations on Propolis Extract

Ethylcellulose microparticles containing propolis ethanolic extract (PE) were prepared by the emulsification and solvent evaporation method. Three ratios of ethylcellulose to PE dry residue value (DR) were tested (1 : 0.25, 1 : 4, and 1 : 10). Moreover, polysorbate 80 was used as emulsifier in the external phase (1.0 or 1.5% w/w). Regular particle morphology without amorphous and/or sticking characteristics was achieved only when an ethylcellulose : DR ratio of 1 : 0.25 and 1.0% polysorbate 80 were used. Microparticles had a mean diameter of 85.83 *μ*m. The entrapment efficiency for propolis of the microparticles was 62.99 ± 0.52%. These ethylcellulose microparticles containing propolis would be useful for developing propolis aqueous dosage forms without the strong and unpleasant taste, aromatic odour, and high ethanol concentration of PE [88].

Pharmaceutical formulations containing poloxamer 407, Carbopol 934P, or gelatin, with ethanolic propolis extract (PE), were designed for the treatment of oral mucosal diseases. PE was produced and its quality was assessed by measuring its specific gravity, pH, weight of dry residue, and total flavonoid content. Monopolymeric and binary polymeric formulations were prepared and their gelling temperature (Tsol/gel), pH, continuous flow rheology, and mucoadhesion were studied. The data obtained on these formulations indicate a potentially useful role in the treatment of oral mucosal diseases [91].

## Figures and Tables

**Table 1:** Geographic origin, main plant sources, and chemical compounds [7].

Sr. no.	Geographic origin	Plant source	Main bioactive compounds	Reference
1	Europe, North America, and nontropic regions of Asia	Populus spp., most often *P. nigra *L.	Polyphenols	[3]
2	Russia	*Betula verrucosa *Ehrh*. *	Polyphenols	[3]
3	Brazil	*Baccharis *spp., predominantly *B. dracunculifolia *DC*. *	Prenylated p-coumaric acids, diterpenic acids	[7]
4	Cuba, Venezuela	*Clusia *spp*. *	Polyprenylated benzophenones	[89]
5	Pacific region (Okinawa, Taiwan)	Unknown	C-prenylflavanones Furofuran lignans	[90]
6	Canary Islands	Unknown	Furofuran lignans	[15]
7	Kenya	Unknown	Polyphenols	[29, 30]
8	Greece and Cyprus	Unknown	Flavonoids, terpenes	[31]

**Table 2:** Different solvents used for the extraction of propolis [32].

Water	Methanol	Ethanol	Chloroform	Dichloromethane	Ether	Acetone
Anthocyanins, starches, tannins, saponins, terpenoids, polypeptides, and lectins	Anthocyanins, terpenoids, saponins, tannins, xanthoxyline, totarol, quassinoids, lactones, flavones, phenones, polyphenols, polypeptides, and lectins	Tannins, polyphenol, polyacetylenes, terpenoids, sterols, and alkaloids,	Terpenoids, flavonoids	Terpenoids, tannins, polyphenols, polyacetylenes, sterols, and alkaloids	Alkaloids, terpenoids, coumarins, and fatty acids	Flavonols

**Table 3:** Geographic origin, activity, and chemical compounds in Indian scenario.

Sr. no.	Geographic region	Activity	Solvent used in extraction	Reference
1	Karnataka	Antibacterial	Petroleum ether, chloroform, ethanol, methanol, and 40% methanol	[24]
2	West Bengal	Antioxidant	Ethanol and water	[18]
3	Gujarat	Antioxidant, antimicrobial	Ethanol, water, petroleum ether, chloroform, ethanol, methanol, and 40% methanol	[16]
4	Madhya Pradesh	Antimicrobial, hepatoprotective	Ethanol	[33]
5	Maharashtra	Antimicrobial, antibacterial	Ethanol	[34]

**Table 4:** Bacteria used in identification of antibacterial activity [32].

Gram-positive	Gram-negative
*Bacillus cereus	*Aeromonas hydrophila
*Bacillus subtilis	*Brucella abortus
*Enterococcus spp. *(Enterococcus faecalis) *	*Corynebacterium sp. *(C. pseudotuberculosis) *
*Micrococcus luteus	*Escherichia coli
*Nocardia asteroids *Rhodococcus equi	*Helicobacter pylori
*Staphylococcus aureus	*Klebsiella pneumoniae
*Staphylococcus spp. *(S. auricularis, S. capitis, S. epidermidis, S. haemolyticus, S. hominis, S. mutans,* and *S. warnerii) *	*Salmonella sp. *(S. enteritidis, S. typhi,* and *S. typhimurium) *
*Streptococcus spp. *(S. cricetus, S. faecalis, S. Pneumonia, S. pyogenes, S. β-haemolyticus, S. mutans, S. sobrinus,* and *S. viridians) *	*Pseudomonas aeruginosa *Proteus mirabilis* *Proteus vulgaris* *Shigella dysenteriae *
**Actinomyces naeslundii * ***Lactobacillus acidophilus **Peptostreptococcus micros	**Actinobacillus actinomycetemcomitans **Capnocytophaga gingivalis **Porphyromonas anaerobius **Prevotella intermedia
	**Fusobacterium nucleatum **Porphyromonas gingivalis **Prevotella melaninogenica
	**Prevotella oralis **Veillonella parvula

*Aerobic bacteria, **anaerobic bacteria.
